# Frequent, geographically structured heteroplasmy in the mitochondria of a flowering plant, ribwort plantain (*Plantago lanceolata*)

**DOI:** 10.1038/hdy.2016.15

**Published:** 2016-03-09

**Authors:** N Levsen, R Bergero, D Charlesworth, K Wolff

**Affiliations:** 1 School of Biology, Newcastle University, Newcastle upon Tyne, UK; 2 Institute of Evolutionary Biology, Ashworth Laboratory, School of Biological Sciences, The University of Edinburgh, Edinburgh, UK

## Abstract

Recent research has convincingly documented cases of mitochondrial heteroplasmy in a small set of wild and cultivated plant species. Heteroplasmy is suspected to be common in flowering plants and investigations of additional taxa may help understand the mechanisms generating heteroplasmy as well as its effects on plant phenotypes. The role of mitochondrial heteroplasmy is of particular interest in plants as cytoplasmic male sterility is controlled by mitochondrial genotypes, sometimes leading to co-occurring female and hermaphroditic individuals (gynodioecy). Paternal leakage may be important in the evolution of mating systems in such populations. We conducted a genetic survey of the gynodioecious plant *Plantago lanceolata*, in which heteroplasmy has not previously been reported, and estimated the frequencies of mitochondrial genotypes and heteroplasmy. Sanger sequence genotyping of 179 individuals from 15 European populations for two polymorphic mitochondrial loci, *atp*6 and *rps*12, identified 15 heteroplasmic individuals. These were distributed among 6 of the 10 populations that had polymorphisms in the target loci and represented 8% of all sampled individuals and 15% of the individuals in those 6 populations. The incidence was highest in Northern England and Scotland. Our results are consistent with geographic differences in the incidence of paternal leakage and/or the rates of nuclear restoration of male fertility.

## Introduction

In genetic studies of flowering plants, it is often assumed that most individuals carry a single complement of mitochondrial genes inherited from the maternal parent. However, increasing numbers of reports from crop (see, for example, Bragin *et al.*, 2012; Szklarczyk *et al.*, 2014) and wild species (see, for example, McCauley *et al.*, 2005; Mandel and McCauley, 2015) have documented the co-occurrence of multiple mitochondrial genotypes within plant cells (termed mitochondrial heteroplasmy), suggesting that it might be common in many angiosperms (Kmiec *et al.*, 2006). Mitochondrial heteroplasmy has important implications for evolution under nuclear and organellar genomic conflict and for the evolution of plant male sterility (Wade and McCauley, 2005; McCauley, 2013; Christie *et al.*, 2015), but very little is known about its effects on plant phenotypes in natural populations. Despite its likely prevalence, heteroplasmy has been documented in populations of only a few plant species (see, for example, Pearl *et al.*, 2009; Mandel and McCauley, 2015). To empirically test the theoretical models of such evolutionary processes we require investigations of heteroplasmy in natural populations.

Two main processes can produce mitochondrial heteroplasmy, mutation and paternal leakage, and once generated heteroplasmy can potentially allow recombination between mitochondrial genotypes (Kmiec *et al.*, 2006). Flowering plants generally exhibit low mitochondrial mutation rates (Wolfe *et al.*, 1987); therefore, paternal leakage is considered the predominant mechanism generating heteroplasmy in angiosperms (McCauley, 2013; Christie *et al.*, 2015). However, species in some genera, *Plantago* included, appear to have very high mitochondrial synonymous substitution rates (Mower *et al.*, 2007) that may reflect high mutation rates. The co-occurrence of nonfunctional mutant cytoplasmic genotypes and functional (nonmutant) genotypes can also arise because nonmutant mitochondrial genomes are necessary for viability. For example, highly pathogenic point mutations in human mitochondrial genes often remain heteroplasmic (Ye *et al.*, 2014). Such heteroplasmy could also be deleterious because of mitochondrial competition, either between functional mitotypes or between functional and nonfunctional mitotypes that could produce metabolic dysfunction (Sharpley *et al.*, 2012; Greiner *et al.*, 2014).

Under strict maternal inheritance, mutations causing loss of male functions are likely to be neutral or beneficial to female fitness and thus can increase mitochondrial transmission to future generations (Lewis, 1942; Greiner *et al.*, 2014). Cytoplasmic male sterility (CMS) polymorphisms can therefore be established (Chase, 2007), and are known in many plant species (reviewed by Schnable and Wise, 1998). The CMS mutant phenotype can be negated by nuclear ‘restorer' alleles; within a species, appearance of a restorer can result in a return to hermaphroditism or lead to long-term gynodioecy with male sterile (female) and hermaphroditic individuals coexisting within populations (Lewis, 1942; Gouyon *et al.*, 1991; Chase, 2007; Touzet and Delph, 2009). Paternal leakage, causing mitochondrial heteroplasmy, can influence the evolution of CMS in two ways: it allows selection on mitochondrial effects on male fitness (Frank, 1989) and can potentially restore male function in the offspring of a CMS mother plant, thereby increasing nuclear gene fitness (Andersson-Ceplitis and Bengtsson, 2002; McCauley, 2013). In the latter case, genotypes that lead to heteroplasmy would be predicted to be favored in populations where rates of nuclear restoration are low. However, little is currently known about the natural incidence of mitochondrial heteroplasmy and its role in the evolution and maintenance of CMS.

We therefore conducted a survey of mitochondrial sequence variants throughout Western Europe in order to estimate frequencies of polymorphism in natural populations of a gynodioecious flowering plant, ribwort plantain (*Plantago lanceolata*), and test for heteroplasmy that has not previously been examined in the species. By combining analyses of sequences with information on sex phenotypes, we also tested whether heteroplasmy is more common in gynodioecious *P. lanceolata* lacking restorers of male fertility than in populations with restorers.

## Materials and methods

### Study system

*P. lanceolata* is a herbaceous flowering plant with a worldwide distribution. The species is obligately outcrossing because of self-incompatibility, and populations are usually gynodioecious (Ross, 1973). Controlled crossing experiments have demonstrated cytonuclear control of sex phenotypes in *P. lanceolata*; three CMS types were identified, with evidence that mitochondrial genotypes differ between them (Van Damme and Van Delden, 1982). Alleles that restore male fertility in the presence of specific CMS types are found at multiple nuclear loci, and interact epistatically (de Haan *et al.*, 1997a). The estimated frequency of male restorer alleles varies among populations and CMS types, being lowest for CMS type-1 (de Haan *et al.*, 1997a).

### Plant collecting and phenotyping

**Figure 1:**
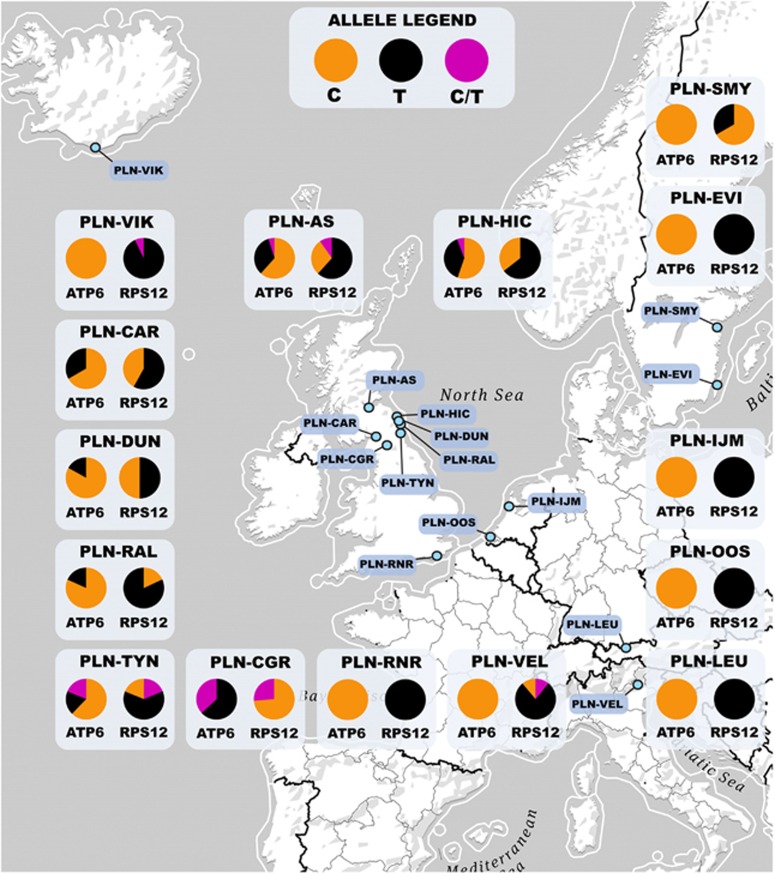
Population locations and mitochondrial genotype frequencies of Western European *P. lanceolata*. The relative proportion of single-nucleotide polymorphism (SNP) genotypes at *atp*6 and *rps*12 (listed in Table 2) are represented in pie charts by color: ‘T' (black), ‘C' (orange) and heteroplasmic ‘C/T' (pink).

Living plants, leaf material or seeds were collected from 15 wild populations of *P. lanceolata* in Western Europe (Table 1 and Figure 1). Sex phenotypes were recorded in the wild at the time of collection or under greenhouse conditions following germination. All individuals represented by dried leaf or living material were genotyped, whereas wild collected seeds were germinated and one plant per seed family was chosen for downstream analyses. Seedlings and living plants were maintained in the Newcastle University growth facilities. Male sterility was identified as the absence of pollen producing anthers on all sexually mature inflorescences. When the phenotype was unambiguous, the trait values ‘male sterile' or ‘hermaphrodite' were assigned; individuals with ambiguous or unknown phenotypes were categorized as ‘undetermined'.

### Sequencing, trace accuracy and genotype calling

DNA was extracted from leaf tissue samples using a modified CTAB (cetyl trimethylammonium bromide) protocol (Wolff, 1996). Partial mitochondrial genome assemblies (constructed from Illumina, San Diego, CA, USA; paired-end reads; N Levsen and K Wolff, unpublished) of eight geographically disparate European *P. lanceolata* individuals were used to design PCR and sequencing primers (Supplementary Table S1) for fragments of two mitochondrial genes (*atp*6 and *rps*12). PCR amplification was conducted in a 20 μl volume consisting of 4.2 μl of 5 × MyTaq Reaction Buffer (Bioline, London, UK), 0.5 μl (10 pM) of each primer, 13.4 μl H_2_O, 0.4 μl MyTaq DNA polymerase and 1 μl of template DNA. The temperature cycling protocol was as follows: 95 °C initial denaturation for 3 min; 35 cycles at 95 °C for 15 s, 52 °C for 15 s and 72 °C for 30 s; a final extension step at 72 °C for 5 min. Sanger sequencing of both DNA strands was performed at the Edinburgh Genomics facility (Edinburgh, UK). The sequence reads were quality trimmed and aligned to the *Mimulus guttatus* mitochondrial reference sequence (GenBank: JN098455.1; Mower *et al.*, 2012) in CLC Genomics Workbench v8.0.2 (CLCbio, Arhus, Denmark). The annotated *M. guttatus* sequence provided a published guide to determining site position and category (for example, synonymous).

### Identifying heteroplasmy

Sanger DNA sequencing at heterozygous or heteroplasmic sites produces two electropherogram trace peaks: one for each segregating nucleotide. The height ratio of those peaks indicated the proportion of each nucleotide in the underlying sample (Roy and Schreiber, 2014). To assess our ability to detect heteroplasmy, we generated mixtures of two alleles of the mitochondrial *atp*6 gene and sequenced them. PCR cloning of *atp*6 (CloneJET PCR cloning kit, Thermo Scientific, Waltham, MA, USA) was performed on an individual heteroplasmic for these variants, revealing a single polymorphism (C/T) at position 443 relative to the *M. guttatus atp*6 start position. Cleaned, quantified (10–20 ng μl^−1^) PCR products representing two clones of each type were mixed to generate the series ranging from 100% T to 100% C, with 10 intermediate mixtures (5:95, 10:90, 20:80, 30:70, 40:60, 60:40, 70:30, 80:20, 90:10 and 95:5). Each mix was bidirectionally sequenced with two replicates. Linear regression of the original allele mix proportions and mean trace peak heights for forward and reverse reads was performed using SigmaPlot v12.5 (Systat Software Inc., San Jose, CA, USA). Based on the results of the allelic mixtures, we set the Sanger trace peak threshold indicating heteroplasmy at a site to >10% of the site's total peak height. Peak height was determined using the ‘Secondary Peak Calling' tool in CLC.

Genotypes at *atp*6 position 443 and *rps*12 position 138 (also polymorphic for C and T) were used to identify heteroplasmic individuals. Individuals with significant peaks for both C and T in both the forward and reverse reads were classified as heteroplasmic.

### Experimental crosses

We performed controlled crosses within *P. lanceolata* to test: (1) whether allele transmission was predominantly maternal; (2) whether mitochondrial heteroplasmy can be inherited; and (3) whether paternal leakage generates new cases of heteroplasmy. The first of these tests was done to check that our sequences were not from nuclear mitochondrial (numt) pseudogenes that are common in plant genomes (Hazkani-Covo *et al.*, 2010). Homoplasmic maternal and paternal plants, as well as one heteroplasmic mother, were chosen from previously genotyped individuals. The plants chosen as parents were grown in adjacent pots and flowering shoots from each were entwined and covered with a paper bag to prevent pollen contamination. The resulting seeds were germinated on moist filter paper and mature progeny were genotyped as above for the *atp*6 and *rps*12 variants.

### Recombination and tests for genotype–phenotype associations

To estimate linkage disequilibrium between *atp*6 and *rps*12 and test for historical recombination between them, we calculated *r*^2^ and the minimum number of recombination events (RM) in DnaSP (Librado and Rozas, 2009). Estimates of *r*^2^ and RM were generated for each population that was polymorphic at both loci, for the geographic region in which both polymorphisms were found (Northern England and Scotland, see Results) and for the entire data set (excluding individuals with missing data). Associations between *atp*6 and/or *rps*12 genotypes and male sterility (excluding plants with ‘undetermined' sex phenotypes) were tested using two-tailed Fisher's exact tests of independence in SigmaPlot. Based on our results, tests were also done separately for individuals from the populations of Northern England and Scotland and for individuals from all populations outside of that region.

## Results

Sanger sequencing was used to assess the incidence of heteroplasmy at two mitochondrial loci: a nonsynonymous Ser/Leu variant in *atp*6 and a synonymous variant in *rps*12. Sequences were generated for 179 wild plants for *atp*6 and 178 individuals for *rps*12. The sequences are in GenBank under accession numbers KU596577–KU596971. Most populations from mainland Europe were monomorphic at both loci, but all Northern English and Scottish populations (hereafter referred to as NES) were polymorphic for both *atp*6 and *rps*12 sequence variants (Table 2 and Figure 1). The *atp*6 with the thymine nucleotide (hereafter denoted by the T-allele) was restricted to the NES region and was generally rarer than the C-allele (except in the CGR population). The *rps*12 C-allele was rare in non-NES populations, although it was the major allele (66.7%) in population SMY/SVL and the commoner allele in one (CGR) of the seven NES populations.

### Sanger trace accuracy

Trace peak height ratios for the *atp*6 thymine nucleotide at position 443 were significantly correlated with the proportions of the T-allelic haplotype in the mixtures sequenced (Supplementary Figures S1a and b for forward and reverse sequencing, respectively) and generally agreed between the two sequencing directions. A single false positive for heteroplasmy was detected (in one reverse read). However, the *r*^2^ values (0.55 and 0.62 for forward and reverse reads, respectively) do not suggest precise allelic quantification. To avoid falsely inferring heteroplasmy when the individual was, in fact, homoplasmic, the threshold for declaring a false peak was set at a minor peak height of 10% of the total for the site being examined. False negatives for heteroplasmy were detected in 26 reads (out of 80 sequences of heteroplasmic mixtures).

### Frequency of heteroplasmy

With the conservative (high) detection threshold just explained, mitochondrial heteroplasmy was detected in 6 of our 15 sampled populations (Table 2 and Figure 1). Heteroplasmy for one or other of the loci was generally found whenever the locus was polymorphic; the only exceptions in Table 2 are populations CGR and VIK. High percentages of plants heteroplasmic for both loci were found in three NES populations. Given that the proportions of mitochondrial genotypes in heteroplasmic plants can vary among cells of a single individual (Woloszynska, 2010), as well as our conservative detection threshold and probable high false negative rate (Supplementary Figure S1), our results probably must underestimate the prevalence of heteroplasmy in these natural populations. Considering only those populations in which heteroplasmy was detected, 15% of plants were heteroplasmic on average.

### Inheritance of mitochondrial alleles in experimental crosses

The *atp*6 and *rps*12 genotypes in the progeny of five crosses between *P. lanceolata* individuals with different mitochondrial genotypes support maternal transmission of the loci (Table 3), confirming that the variants are in mitochondrial genes. In addition, heteroplasmy at *atp*6 and *rps*12 was transmitted from the maternal parent of the CGR18 × IJM11.2 cross to three of the four progeny (the reciprocal cross was not performed). Our Illumina paired-end read data (see section on sequencing methods) for *atp*6 in this maternal plant gives an allele ratio estimate of ∼76% T-allele (53T-allele reads versus 17 C).

### Recombination

In the NES populations as a whole, both loci are polymorphic and all four haplotypes were found, whereas the non-NES populations included only the C-T and C-C (*atp*6-*rps*12) haplotypes at very different frequencies from those in the NES populations (Table 4). Shared haplotypes exhibited very different frequencies between the two data sets. Linkage disequilibrium between the *atp*6 and *rps*12 genes was significant in the pool of NES individuals (in these populations, both loci are polymorphic) and in five of the six individual NES populations that could be tested. Estimates from the NES population data set yield at least one historical recombination event between the genes (RM=1), but all individual populations included only three of the four possible haplotypes and, therefore, yielded zero RM estimates.

### Genotype–phenotype association

Male sterility data were obtained for 89 individuals, including 7 heteroplasmic individuals (out of 15 in the total data set); all 7 were from 2 NES populations, TYN and CGR (4 were hermaphroditic and 3 were male sterile, see Supplementary Table S2). The male sterile individuals could be identified phenotypically as either CMS type-1 or type-3 (see Supplementary Table S2; Van Damme and Van Delden, 1982). Associations between the mitochondrial genotypes and sex phenotypes were tested separately for *atp*6 and *rps*12, as there is evidence that the loci recombine (Table 4). In the NES data, both loci showed associations, with the *atp*6 C and *rps*12 T-alleles significantly overrepresented among male sterile individuals (Table 5). In contrast, in the non-NES sample, with only *rps*12 polymorphic, no association was found with the T-allele.

## Discussion

Our results show that gynodioecious populations of *P. lanceolata* contain single-nucleotide polymorphism sequence variants in mitochondrial genes, including both synonymous and nonsynonymous variants. Previous studies of mitochondrial genotypes in this species used anonymous variants detected by restriction enzymes (Rouwendal *et al.*, 1987; de Haan *et al.*, 1997b). We also confirmed, in further populations of this plant, these authors' tentative previous observations of associations between mitochondrial genotypes and male sterility in Dutch populations. Finally, we describe the first evidence of mitochondrial heteroplasmy in this plant.

Even with our moderate sample sizes, and just two loci, we identify at least ~8% (15 of 179) of the sampled individuals as heteroplasmic at one of the mitochondrial loci sequenced or, in 9 individuals, both (Table 2 and Supplementary Table S2). This percentage is lower than detected in natural populations of *Silene vulgaris* (~15% Pearl *et al.*, 2009) and *Daucus carota* (30% Mandel and McCauley, 2015), possibly because the quantitative real-time PCR detection method used in those studies is more sensitive.

Heteroplasmic *P. lanceolata* individuals were found in 6 of the 15 populations sampled, including 4 populations from NES (Table 2 and Figure 1). Recombinant haplotypes were found in all NES populations but CGR, even in the two (DUN and CAR) in which heteroplasmy was not detected, indicating that recombination (Table 4) occurs, or has occurred, in their history. One cannot therefore assume that the *P. lanceolata* mitochondrial genome is nonrecombining when studying its sequence diversity (McCauley, 2013).

Plant mitochondrial heteroplasmy probably results primarily from paternal leakage and this has indeed been detected in family studies of *S. vulgaris* (Bentley *et al.*, 2010) and in wild populations of *Daucus* (Mandel and McCauley, 2015); however, in *Daucus* a consistent deficit of the T variant in the *atp*9 gene is unexpected unless other processes also operate. Our experimental crosses (Table 3) provided no direct evidence for paternal leakage for either *atp*6 or *rps*12 alleles in *P. lanceolata*, but larger families should be tested. We did, however, find indirect evidence of paternal leakage: all four two-locus haplotype combinations were present in natural populations (Table 4). The generation of all four haplotype combinations between two two-allele loci requires either recombination between two divergent co-occurring mitochondrial genomes (Hudson and Kaplan, 1985) or else convergent evolution (McCauley and Olson, 2008). The independent appearance of the same variant by mutation occurring multiple times within a population is much less likely than a recombination event, particularly if the mutation rate in plant mitochondria is low. Although an elevated ancestral mitochondrial synonymous substitution rate has been inferred in the *Plantago* lineage (Mower *et al.*, 2007), rates vary greatly among *Plantago* species and the rate estimated for *P. lanceolata* is among the lowest in the genus (Cho *et al.*, 2004). However, we cannot exclude convergent mutation as an explanation for our results, given that none of our local population samples included all four haplotype combinations and that the T-T haplotype was represented by only one individual in the data set (Tables 2 and 4).

Once heteroplasmy is established, it may persist for multiple generations by maternal transmission, which we detect in *P. lanceolata* (Table 3). Nevertheless, it is expected to diminish over the generations because of the bottleneck in the egg cells each generation, and this could explain the absence of heteroplasmy in one offspring of CGR18 (Table 3). Stochastic losses may account for the highly variable frequencies of heteroplasmy in the different populations, with much higher frequencies in the CGR and TYN populations than in the other populations that exhibit polymorphisms. Alternatively, the variable frequencies could be because of selection.

A strong association between a mitochondrial genotype and the sex phenotype is evidence that nuclear genes restoring male fertility are not common in a population. We observe associations with the variants in both genes studied here, *atp*6 and *rps*12, in the NES *P. lanceolata* populations (Table 5). In contrast, despite a similar abundance of male sterile plants in our samples from the non-NES populations (both ∼40%, see Supplementary Table S2), the *atp*6 gene is not polymorphic, and the presence of the C versus T variant of the *rps*12 gene does not predict plants' sex phenotypes in these populations (Table 5). Moreover, the *rps*12 C variant is rare in most populations in both sex types among the plants we phenotyped (Table 2). Nuclear restoration of male fertility therefore probably occurs oftener in those populations than in the NES region.

If one or more mitochondrial haplotypes carry a sterility factor, paternal leakage of a nonsterility haplotype could be an alternative mechanism that restores male fertility (Andersson-Ceplitis and Bengtsson, 2002; Wade and McCauley, 2005). Genotypes that are transmitted via paternal leakage and favored for their ability to restore male fertility should then occur most frequently in populations in which the rate of nuclear male restoration is low for at least one sterilizing element. However, given the absence of any mitochondrial variant associated with CMS in the non-NES populations, the lower frequency of mitochondrial heteroplasmy may simply be because of low diversity rather than a low rate of nuclear male restoration.

The differences between the two sets of populations studied here suggest the loss of a male fertile mitochondrial type (with the T-C haplotype) from the non-NES populations, rather than the spread of the male fertile haplotype in NES populations. Table 5 shows that the allele frequencies at both loci are similar in females in both sets of populations, whereas in hermaphrodites, the *atp*6 T variant has a much higher frequency in the NES populations (and is much more common than in females); the same is true for the *rps*12 C variant. This suggests a loss of the T-C haplotype, specifically in non-NES population hermaphrodites (see Table 4), and fixation of a male sterility haplotype with the C variant in *atp*6 and the synonymous *rps*12 T variant (the most common haplotype in females in NES populations, see Table 5). Supplementary Table S2 shows that, of the 24 NES females genotyped, 19 had the C-T haplotype, 3 were heteroplasmic (genotypes C/T-C/T) and only 2 had T-C. In contrast, of the 29 NES hermaphrodites genotyped, 14 had T-C, only 10 had C-T, 3 were heteroplasmic at both loci and the C-C and T-T haplotypes were each carried by one individual. The T-C haplotype is thus associated with male fertility and C-T with male sterility, although the association is not complete (consistent with our evidence above that recombination occurs in this plant's mitochondrial genome). The possibility that the C-T haplotype (associated with male sterility in the NES region where restoration is infrequent) has become fixed in the non-NES populations conforms to the theoretical prediction (Frank, 1989) that CMS polymorphisms may often be ephemeral, that polymorphism may be lost once a restorer appears, and that this causes fixation of the CMS mitochondrial genotype, in an ‘epidemic' or ‘arms race' scenario. Uneven rates of restorer activity across populations have been previously documented in *P. lanceolata* (de Haan *et al.*, 1997a); however, the differences reported here may have been caused or exacerbated by long-term gene flow restriction between mainland Europe and the northern British Isles (Searle *et al.*, 2009). Whether the ‘epidemic' scenario is likely to have happened in the *P. lanceolata* populations should be further tested in the future by comparing diversity of mitochondrial sequences with that of nuclear genes, including sequences from related outgroup species, in order to take account of possible mutation rate differences (Ingvarsson and Taylor, 2002).

At present, the high detection threshold and low precision of the Sanger genotyping method precludes additional investigations of allele ratios within individual *P. lanceolata* plants or allelic dosage effects (Stewart and Chinnery, 2015) on sex phenotypes. Neither the *atp*6 nor the *rps*12 locus was associated with a restriction site and the flanking sequence for both loci made allele-specific priming unreliable. However, now that it is known that at least some *P. lanceolata* populations are heteroplasmic, it should be possible to identify heteroplasmic variants in other loci that are amenable to more sensitive approaches such as restriction site genotyping, quantitative real-time PCR or quantitative genotyping via deep sequencing. Further investigations of mitochondrial heteroplasmy in *P. lanceolata* will depend on sensitive and consistent allele quantification and include: direct tests of paternal leakage using expanded experimental crossing designs, of gene-by-gene and whole mitochondrial genome associations with male sterility and of the threshold heteroplasmy ratio for phenotypic effects.

## Data archiving

DNA sequences are available at GenBank (KU596577—KU596971).

## Figures and Tables

**Table 1:** *Plantago lanceolata* collection locations in Western Europe

*Population*	*Location*	*Latitude*	*Longitude*	*MS*	*H*	*UD*
AS	Edinburgh (GB, NES)	55.94°	−3.16°	4	7	10
CGR	Cow Green (GB, NES)	54.67°	−2.29°	2	6	11
CAR	Carlisle (GB, NES)	54.9°	−2.94°	4	8	0
DUN	Craster (GB, NES)	55.48°	−1.59°	3	1	2
HIC	Holy Island (GB, NES)	55.67°	−1.8°	4	4	10
RAL	Alnmouth (GB, NES)	55.38°	−1.61°	4	0	7
TYN	Tynemouth (GB, NES)	55.02°	−1.42°	5	4	7
RNR/SSW	Hastings (GB)	50.86°	0.6°	5	7	2
VIK	Vik (Iceland)	63.42°	−19.00°	0	0	15
EVI	Oland (Sweden)	56.66°	16.64°	0	1	3
SMY/SVL	Gryt (Sweden)	58.17°	16.85°	0	2	4
IJM	Ijmuiden (The Netherlands)	52.46°	4.61°	3	2	3
OOS	Oostkapelle (The Netherlands)	51.57°	3.55°	4	1	9
LEU	Leutasch (Austria)	47.37°	11.14°	0	6	1
VEL	Feltre (Italy)	46.02°	11.9°	1	3	5

Abbreviations: H, hermaphroditic; MS, male sterile; NES, a population from Northern England or Scotland; UD, undetermined.

The number of sampled MS, H and UD individuals is provided for each population in the study.

**Table 2:** Raw counts and percentages of *atp*6 and *rps*12 mitochondrial genotypes across 15 populations of *Plantago lanceolata*

	*Total sample*	*atp6*						*rps12*					
		*C*		*T*		*C/T*		*C*		*T*		*C/T*	
		*Count*	*%*	*Count*	*%*	*Count*	*%*	*Count*	*%*	*Count*	*%*	*Count*	*%*
*NES region*													
AS	21	13	61.9	7	33.3	1 (0)	4.8	6	28.6	13	61.9	2 (1)	9.5
CGR	19	0	0	12	63.2	7 (2)	36.8	14	73.7	0	0	5 (0)	26.3
CAR	12	8	66.7	4	33.3	0	0	5	41.7	7	58.3	0	0
DUN	6	5	83.3	1	16.7	0	0	3	50	3	50	0	0
HIC	18	10	55.5	7	38.9	1 (1)	5.6	6	35.3	11	64.7	0	0
RAL	11	9	81.8	2	18.2	0	0	2	18.2	9	81.8	0	0
TYN	16	10	62.5	3	18.8	3 (0)	18.8	3	18.8	10	62.5	3 (0)	18.8

*Non-NES region*													
RNR/SSW	14	14	100	0	0	0	0	0	0	14	100	0	0
VIK	14	14	100	0	0	0	0	0	0	13	92.9	1 (1)	7.1
EVI	4	4	100	0	0	0	0	0	0	4	100	0	0
SMY/SVL	6	6	100	0	0	0	0	4	66.7	2	33.3	0	0
IJM	8	8	100	0	0	0	0	0	0	8	100	0	0
OOS	14	14	100	0	0	0	0	0	0	14	100	0	0
LEU	7	7	100	0	0	0	0	0	0	7	100	0	0
VEL	9	9	100	0	0	0	0	1	11.1	7	77.8	1 (1)	11.1

Abbreviation: NES, a population from Northern England or Scotland.

Numbers of individuals genotyped for at least one locus are listed in the ‘Total Sample' column. Genotype percentages are calculated based on the total number of individuals sequenced at the specific locus. The numbers of individuals that are only heteroplasmic at a single given locus are listed in parentheses next to the ‘C/T' genotype count numbers.

**Table 3:** Genotypes in crosses between *Plantago lanceolata* plants with different mitochondrial genotypes

*Parents*	*Cross*	*atp6*	*rps12*	*Reciprocal cross*	*atp6*	*rps12*
CGR12 and SMY14	CGR12 maternal	T	C	SMY14 maternal	C	T
	SMY14 pollen donor	C	T	CGR12 pollen donor	T	C
	Offspring	All 5T	All 5 C	Offspring	All 6 C	All 6T
SVL04 and VEL16	SVL04 maternal plant	C	C	SVL04 maternal plant	C	T
	VEL16 pollen donor	C	T	VEL16 pollen donor	C	C
	Offspring	Both C	Both C	Offspring	Both C	Both T
CGR18 and IJM11.2	CGR18 maternal plant	C/T	C/T			
	IJM11.2 pollen donor	C	T			
	Offspring	One T, 3 C/T	One C, 3 C/T			

Mitochondrial single-nucleotide polymorphism (SNP) genotypes for parents and progeny are reported for each variable site within two gene fragments. A forward slash separates nucleotides that co-occur in significant proportion (⩾10%) at a given site within an individual; the co-occurrence of these nucleotides is considered evidence of heteroplasmy.

**Table 4:** Estimates of mitochondrial recombination and linkage disequilibrium in *Plantago lanceolata*

*Population*	*Haplotypes for atp6 and rps12*				*RM*	r^*2*^	*Fisher's exact test* P*-value*
	*T-T*	*T-C*	*C-T*	*C-C*			
AS (NES)	0	6	13	0	0	1.00	<0.001
CAR (NES)	0	4	7	1	0	0.70	<0.05
DUN (NES)	0	1	3	2	0	0.20	NS
HIC (NES)	1	6	9	0	0	0.77	<0.001
RAL (NES)	0	2	9	0	0	1.00	<0.05
TYN (NES)	0	3	10	0	0	1.00	<0.01
CGR (NES)	0	12	0	0	—	—	—
NES populations pooled	1	34	51	3	1	0.82	<0.001
RNR/SSW	0	0	14	0	—	—	—
VIK	0	0	13	0	—	—	—
EVI	0	0	4	0	—	—	—
SMY/SVL	0	0	2	4	0	—	—
IJM	0	0	8	0	—	—	—
OOS	0	0	14	0	—	—	—
LEU	0	0	7	0	—	—	—
VEL	0	0	7	1	0	—	—
Non-NES populations pooled	0	0	69	5	0	—	—
Total data set	1	34	120	8	1	0.73	<0.001

Abbreviations: NES, a population from Northern England or Scotland; NS, not significant.

RM estimates the minimum number of historical recombination events between the pair of single-nucleotide polymorphism (SNPs), one in each gene (*atp6* pos. 443 and *rps*12 pos. 138), and *r*^2^ is a measure of linkage disequilibrium. The *P*-values reported from Fisher's exact tests indicate the significance of linkage disequilibrium between the two polymorphic sites. The symbol ‘—' denotes a value that could not be calculated because of absence of the *atp*6 polymorphism in the population.

**Table 5:** Tests of independence between mitochondrial genotype and sex phenotype in *Plantago lanceolata*

*Data set*		*Phenotype and genotype*				P-*value*
		*Male sterile*		*Hermaphrodite*		
		*T*	*C*	*T*	*C*	
*atp*6 NES	Observed count	2	20	14	11	<0.001
	Expected	7.49	14.51	8.51	16.49	
*rps*12 NES	Observed count	19	2	11	14	0.002
	Expected	13.7	7.3	16.3	8.7	
*atp*6 non-NES	Observed count^a^	0	12	0	22	**—**
*rps*12 non-NES	Observed count	12	1	21	1	1.00
	Expected	12.26	0.74	20.74	1.26	

Abbreviation: NES, a population from Northern England or Scotland.

The ‘NES' data set includes only individuals sampled from populations located in Northern England or Scotland. In this pool of plants, up to 47% of individuals were male sterile, versus up to 35% in the other set of plants.

a The test could not be performed for the *atp*6 non-NES populations because the C-allele was fixed in these populations.
