# Supplementary material for: Frequent, geographically structured heteroplasmy in the mitochondria of a flowering plant, ribwort plantain (Plantago lanceolata)
Source: Heredity (Edinb). 2016 Mar 9;117(1):1–7. doi: 10.1038/hdy.2016.15 (PMC4901351; doi:10.1038/hdy.2016.15)
Supplement: Supplementary Table 1 [file hdy201615x1.pdf]

Table S1. *Plantago lanceolata* PCR and sequencing primers for two mitochondrial gene regions.

Primer Name	Primer Sequence	Primer Type	Primer Direction	Gene Region
pr17.atp6.lanc.for1	5’-TGAAGGAAGCTCTTTCATTGG-3’	PCR	Forward	*atp*6
pr18.atp6.lanc.rev1	5’-GAGCGCAAAAAGAAGTACTTTGA-3’	PCR	Reverse	*atp*6
pr122.ATP6.Fseq	5’-CCCCTTGAGCAATTTTCC-3’	Sequence	Forward	*atp*6
pr123.ATP6.Rseq	5’-TACACCTAATTCGAGACC-3’	Sequence	Reverse	*atp*6
pr60.rps12.F	5’-CTACTAATCAATTAATTCGGGATGG-3’	PCR/Sequence	Forward	*rps*12
pr61.rps12.R	5’-CATATTTTGATCTTCCTCTTCTTCG-3’	PCR/Sequence	Reverse	*rps*12
