# Supplementary material for: Frequent, geographically structured heteroplasmy in the mitochondria of a flowering plant, ribwort plantain (Plantago lanceolata)
Source: Heredity (Edinb). 2016 Mar 9;117(1):1–7. doi: 10.1038/hdy.2016.15 (PMC4901351; doi:10.1038/hdy.2016.15)
Supplement: Supplementary Table 2 [file hdy201615x2.pdf]

Table S2. Raw mitochondrial genotype and sex phenotype data for sampled Western European individuals of *Plantago lanceolata*.

Region	Population	Individual	Phenotype	Genotype	
				*atp*6	*rps*12
NES					
	AS	AS07	UD	C	T
		AS47	UD	T	C
		AS49	UD	T	C
		AS73	UD	T	C/T
		AS77	UD	C/T	C/T
		AS78	UD	T	C
		AS90	UD	T	C
		AS104	UD	C	T
		AS108	UD	C	T
		AS116	UD	C	T
		EDI01	MS1_MS3	C	T
		EDI02	MS1_MS3	C	T
		EDI03	MS1_MS3	C	T
		EDI04	MS1_MS3	C	T
		EDI05	H	C	T
		EDI06	H	C	T
		EDI07	H	T	C
		EDI09	H	C	T
		EDI10	H	C	T
		EDI11	H	C	T
		EDI12	H	T	C
	CGR	CGR05	UD	C/T	C
		CGR07	UD	T	C
		CGR08	UD	T	C
		CGR09	UD	T	C
		CGR11	H	T	C
		CGR12	H	T	C
		CGR13	UD	T	C
		CGR16	UD	C/T	C/T
		CGR18	H	C/T	C/T
		CGR19	H	C/T	C
		CGR20	UD	T	C
		CGR21	H	C/T	C/T
		CGR23	UD	T	C
		CGR29B	MS1	C/T	C/T
		CGR32	UD	T	C
		CGR36	UD	T	C
		CGR39	UD	T	C
		CGR40B	MS1	C/T	C/T
		CGR44	UD	T	C
	CAR	CAR01	MS1_MS3	C	T
		CAR02	MS1_MS3	C	T

Region	Population	Individual	Phenotype	Genotype	
				*atp*6	*rps*12
		CAR03	H	C	T
		CAR04	H	C	T
		CAR05	H	C	T
		CAR06	H	T	C
		CAR07	MS1_MS3	C	T
		CAR08	H	T	C
		CAR09	MS1_MS3	C	T
		CAR10	H	T	C
		CAR11	H	C	C
		CAR12	H	T	C
	DUN	DUN06	MS1	C	T
		DUN07	MS1	C	T
		DUN12	H	T	C
		DUN13	MS1	C	T
		CRA4.2B	UD	C	C
		CRA5.1C	UD	C	C
	HIC	HIC02	UD	T	C
		HIC05	UD	C/T	T
		HICnonr	MS1	C	T
		HIP101	H	T	C
		HIP102	MS1	C	T
		HIP103	UD	T	C
		HIC07	UD	C	T
		HIC08	UD	C	T
		HIC09	MS1	C	-
		HIC10	UD	C	T
		HIC11	UD	C	T
		HIC12	UD	C	T
		HIC13d	UD	C	T
		HIC14	UD	C	T
		HIC17	UD	T	C
		HIC18	H	T	C
		HIC19	H	T	T
		HIC20.101	H	T	C
	RAL	RAL01	UD	C	T
		RAL02	UD	C	T
		RAL03	UD	C	T
		RAL04	UD	C	T
		RAL11	UD	T	C
		RAL14	UD	T	C
		RAL18	UD	C	T
		RAL25	MS1	C	T
		RALMS2	MS1	C	T
		RALMS3	MS3	C	T
		SGSnr2	MS1	C	T
	TYN	TYN01	UD	C	T

Region	Population	Individual	Phenotype	Genotype	
				*atp*6	*rps*12
		TYN02	UD	C	T
		TYN03	UD	C	T
		TYN04	UD	C	T
		TYN05	UD	C	T
		TYN06	UD	C	T
		TYN07	UD	C/T	C/T
		TYN08	H	T	C
		TYN09	H	C	T
		TYN10	H	C	T
		TYN11	H	C/T	C/T
		TYN12	MS1_MS3	T	C
		TYN13	MS1_MS3	T	C
		TYN14	MS1_MS3	C	T
		TYN16	MS1_MS3	C	T
		TYN17	MS1_MS3	C/T	C/T
non-NES					
	RNR/SSW	RNR01	H	C	T
		RNR03	MS1	C	T
		RNR04	H	C	T
		RNR06	H	C	T
		SSW01	H	C	T
		SSW03	MS1	C	T
		SSW05	UD	C	T
		SSW06	H	C	T
		SSW07	UD	C	T
		SSW09	MS1	C	T
		SSW13	H	C	T
		SSW14	H	C	T
		SSW15	MS1	C	T
		SSW16	MS1	C	T
	VIK	VIK01	UD	C	T
		VIK03	UD	C	T
		VIK04	UD	C	T
		VIK05	UD	C	T
		VIK07	UD	C	T
		VIK08	UD	C	T
		VIK09	UD	C	T
		VIK10	UD	C	T
		VIK11	UD	C	T
		VIK12	UD	C	T
		VIK13	UD	C	T
		VIK14	UD	C	T
		VIK15	UD	C	C/T
		VIK16	UD	C	T
	EVI	EVI04	UD	C	T
		EVI05	UD	C	T

Region	Population	Individual	Phenotype	Genotype	
				*atp*6	*rps*12
		OLA04	H	C	T
		OLA05	UD	C	T
	SMY/SVL	SMY03	H	C	C
		SMY14	UD	C	C
		SVL01	H	C	T
		SVL02	UD	C	T
		SVL03	UD	C	C
		SVL04	UD	C	C
	IJM	IJM01.2	MS1	C	T
		IJM02.3	H	C	T
		IJM04.1	H	C	T
		IJM05	MS1	C	T
		IJM08	MS1	C	T
		IJM10	UD	C	T
		IJM11.2	UD	C	T
		IJM16.101	UD	C	T
	OOS	OOS01	UD	C	T
		OOS03	MS1	C	T
		OOS04	UD	C	T
		OOS05	UD	C	T
		OOS07	UD	C	T
		OOS09	UD	C	T
		OOS12	UD	C	T
		OOS13	H	C	T
		OOS15e	MS1	C	T
		OOS17	MS1	C	T
		OOS19	UD	C	T
		OOS20	UD	C	T
		OOS21	UD	C	T
		OOS22d	MS3	C	T
	LEU	LEU07	UD	C	T
		LEU08	H	C	T
		LEU09	H	C	T
		LEU10	H	C	T
		LEU11	H	C	T
		LEU12	H	C	T
		LEU18	H	C	T
	VEL	VEL02	H	C	T
		VEL04	H	C	T
		VEL05	UD	C	T
		VEL06	UD	C	T
		VEL07	H	C	T
		VEL09	MS3	C	C
		VEL14	UD	C	T
		VEL16	UD	C	T
		VEL17	UD	C	C/T

The populations are separated into the two regions described in the main text: Northern England and Scotland (NES) and elsewhere in Western Europe (non-NES). A dash indicates missing data. Sex phenotypes are represented by ‘MS’ for male sterile, ‘H’ for hermaphrodite, and ‘UD’ for undetermined. The two previously recognized types of male steriles, MS1 and MS3, are distinguished. The underscore between MS types indicates that the sex phenotype may be of either type or an intermediate. The forward slash between nucleotides indicates a heteroplasmic genotype.
