# Supplementary material for: Frequent, geographically structured heteroplasmy in the mitochondria of a flowering plant, ribwort plantain (Plantago lanceolata)
Source: Heredity (Edinb). 2016 Mar 9;117(1):1–7. doi: 10.1038/hdy.2016.15 (PMC4901351; doi:10.1038/hdy.2016.15)

Figure S1. Mitochondrial *atp*6 T-allele quantification using Sanger trace peaks in *P. lanceolata*. Average (two replicates) measured proportional peak heights for the T nucleotide are plotted against the T proportion in allelic mixtures (labeled Expected) for both forward **(a)** and reverse **(b)** sequencing reads.

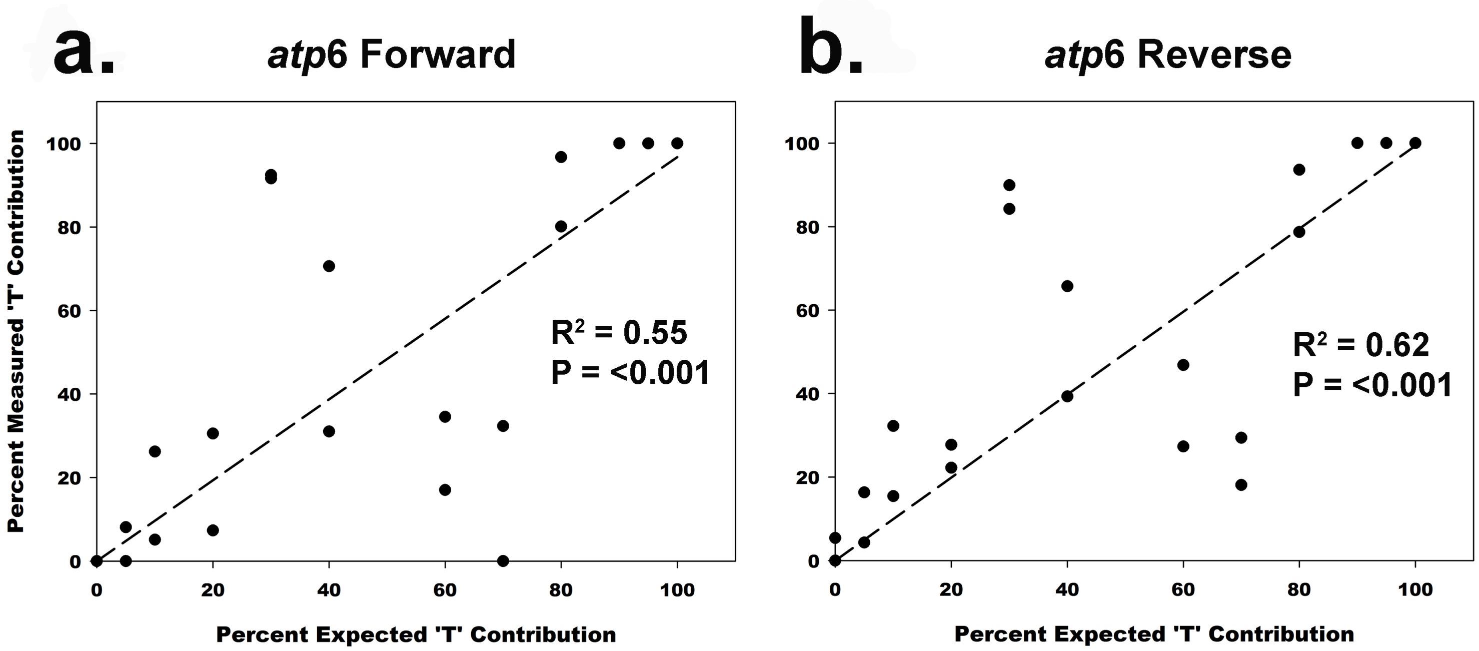

Supplement: Supplementary Figure 1 [file hdy201615x3.pdf]
